# Performant Composite Materials Based on Oxide Semiconductors and Metallic Nanoparticles Generated from Cloves and Mandarin Peel Extracts

**DOI:** 10.3390/nano10112146

**Published:** 2020-10-28

**Authors:** Irina Zgura, Monica Enculescu, Cosmin Istrate, Raluca Negrea, Mihaela Bacalum, Liviu Nedelcu, Marcela Elisabeta Barbinta-Patrascu

**Affiliations:** 1 National Institute of Materials Physics, Atomistilor 405A, 077125 Magurele, Romania; mdatcu@infim.ro (M.E.); cosmin.istrate@infim.ro (C.I.); raluca.damian@infim.ro (R.N.); nedelcu@infim.ro (L.N.); 2 Horia Hulubei National Institute for Physics and Nuclear Engineering (IFIN-HH), Department of Life and Environmental Physics, 077125 Bucharest-Magurele, Romania; bmihaela@nipne.ro; 3 Faculty of Physics, University of Bucharest, 405 Atomistilor Street, P.O. Box MG-11, 077125 Bucharest-Magurele, Romania

**Keywords:** ZnO–Ag composites, “green” synthesis, antibacterial activity, cytotoxicity

## Abstract

In this work, the metal and semiconducting nanoparticles (AgNPs, ZnONPs and AgZnONPs) were phyto-synthesized using aqueous vegetal extracts from: *Caryophyllus aromaticus* L. (cloves) and *Citrus reticulata* L. (mandarin) peels. The morphological, structural, compositional, optical and biological properties (antibacterial activity, and cytotoxicity) of the prepared composites were investigated. The most effective sample proved to be AgZnONPs, derived from cloves, with a minimum inhibitory concentration (MIC) value of 0.11 mg/mL and a minimum bactericidal concentration (MBC) value of 2.68 mg/mL. All the other three composites inhibited bacterial growth at a concentration between 0.25 mg/mL and 0.37 mg/mL, with a bactericidal concentration between 3 mg/mL and 4 mg/mL. The obtained composites presented biocidal activity against *Staphylococcus aureus*, and biocompatibility (on human fibroblast BJ cells) and did not damage the human red blood cells. Additionally, an important result is that the presence of silver in composite materials improved the bactericidal action of these nanomaterials against the most common nosocomial pathogen, *Staphylococcus aureus*.

## 1. Introduction

The design of new antibacterial systems is of real interest today, in the context of nosocomial infections producing many victims, as bacteria become increasingly resistant to antibiotics. Advances in nanotechnology, the science dealing with the designing, manipulation, characterization, controlling and understanding, in a multidisciplinary manner, of nano-scaled objects [1] are helping in the fight against pathogens. Nano-scaled inorganic compounds like silver and zinc oxide exhibit antibacterial activity at a very low dose due to their high surface area to volume ratio which confer them interesting features [2]. Silver nanoparticles (AgNPs) have been used for a long time as antimicrobials, due to their interesting physical, chemical and biological properties, making them suitable for applications in industry and biomedical fields [3]. Due to non-penetrating property to human skin, AgNPs have been used as safe preservatives in different cosmetic products [4]. Like silver, ZnO is known for its antimicrobial activity from immemorial times [5], being used during the regime of Pharaohs (in 2000 BC) in many ointments for disease, treatment of injuries and boils. The US FDA has enlisted ZnO as a GRAS (generally recognized as safe) metal oxide [6]. Nowadays, ZnO is used in cosmetics, in sunscreen lotions, optoelectronic devices, solar cells, photo-catalysis [7]. 

In the last two decades, ZnONPs have become some of the most popular materials in biomedical applications (anticancer, drug delivery, antibacterial, wound healing, diabetes treatment, etc.) due to their excellent biocompatibility, low-cost, and low toxicity [8,9]. The research team of Arshad [10] reported the preparation of ZnONPs embedded in a biodegradable bandage for protection of postoperative surgical site infection. 

Taking into account the resistance to antibiotics, the idea of obtaining efficient biocidal agents led to the realization of AgZnONPs biocomposites with increased antimicrobial efficiency, being exceptional candidates for wound dressings [11]. Thus, Naseri et al. [12] highlighted the antimicrobial potency of hybrids based on AgZnONPs and chitosan, and the effectiveness of collagen–chitosan–AgZnONPs in wound healing and re-epithelialization. AgNPs, ZnONPs and AgZnONPs were found to be effective antibacterial and antifungal agents; the antimicrobial activity of AgZnONPs has been reported to be dependent upon size, morphological properties and silver amount [13].

Over recent decades, considerable attention has been given to the “green” synthesis of nanoparticles (NPs), especially to plant-mediated routes to obtain nanomaterials with low-cost, biocompatibility, and enhanced biological activities. Phyto-synthesized metallic and bimetallic nanoparticles are suitable for bio-applications, due to their improved biocompatibility and bio-performances (antimicrobial, antioxidant and anti-proliferative activities) [14,15,16,17]. The use of vegetal extracts to generate valuable nanomaterials is a preferred “green” approach, considering that plants are a cheap and abundant source in nature and a precious reservoir of bioactive compounds. Phytogenic AgNPs and their biocomposites have always attracted the attention of the scientific community, due to their interesting properties: antitumor [18], antioxidant and antimicrobial [15,19,20] activities. A broad variety of plant extracts have been used for the biosynthesis of ZnONPs such as the leaves of *Aloe barbadensis*, *Plectranthus amboinicus*, the peel of *Nephelium lappaceum*, the root extract of *Polygala tenuifolia*, the rhizome extract of *Zingiber officinale*, the flower extract of *Trifolium pratense*, and so on [2,6]. Abbasi et al. developed a “green” synthesis method of ZnONPs from different tissues of *Silybum marianum*, with antioxidant, antibacterial and anti-proliferative properties [21]. 

Considering the valuable properties of “green” AgNPs and ZnONPs, it is expected that “green” AgZnONPs biocomposites have improved bioactivities as compared to their components. Besides their interesting features (antioxidant and antibacterial properties, photocatalytic activity), very few studies exist in the scientific literature regarding the “green” synthesis of AgZnONPs. In this paper, the metal and semiconducting nanoparticles (AgNPs, ZnONPs and AgZnONPs) were phyto-synthesized using aqueous vegetal extracts from: *Caryophyllus aromaticus* L. (cloves) and *Citrus reticulata* L. (mandarin) peels. The choice of these plants is due to their multiple health benefits. Thus, *Caryophyllus aromaticus* (cloves), a precious spice with aromatic flower buds, contains many bioactive compounds such as: essential oils, phenolic acids, and flavonoids that are responsible for antimicrobial, anti-inflammatory and antioxidant properties [14,22]. Another natural resource used in this study, to generate metallic nanoparticles (NPs), are mandarin peels that are a low-priced source containing many natural bioactive compounds: polyphenols, flavonoids, ascorbic acid, carotenoids, and essential oils [23,24]. Moreover, *Citrus* peels contain large amounts of pectin, another valuable bio-compound, a polysaccharide widely used in biomedical fields [25,26], and in the food industry [27]. To our knowledge, up to now there is no report on the “green” synthesis of AgZnONPs composites using aqueous vegetal extracts of *Caryophyllus aromaticus* L. flower buds and *Citrus reticulata* L. peels, and their effects on human fibroblast cells and on *Staphylococcus aureus* pathogenic bacterium. 

## 2. Materials and Methods 

The present study is focused on the ecological synthesis of AgZnO composites and their complex characterization. The synthesis of composites consists of two steps: (i) preparation of Ag nanoparticles (AgNPs). The bioreduction of silver ions was observed visually, by changing the color of the extracts after the addition of 1 mM AgNO_3_ solution and (ii) AgNPs suspension was further used in the generation of ZnO microparticles by reaction between Zn (NO_3_)_2_ and NaOH.

### 2.1. Samples Preparation

The chemical reagents AgNO_3_, Zn (NO_3_)_2_·6H_2_O and NaOH are of Merck origin (Darmstadt, Germany) and have been used without any further purification.

(i) **Preparation of extracts**
  (a) the cloves extract
    A quantity of 50 g of the cloves powder was introduced into 250 mL of hot distilled water. The suspension was boiled for 5 min, then cooled to room temperature and filtered using Whatman filter paper no.1. The aqueous extract was used in subsequent experiments to obtain AgNPs and ZnO-based structures.
  (b) mandarin peels extract
    A quantity of 50 g of mandarin peels (previously washed with distilled water) was introduced into 250 mL of hot distilled water. The suspension was boiled for 45 min, then cooled to room temperature and filtered using filter paper (Whatman filter paper no.1). The aqueous mandarin extract was used in subsequent experiments to obtain AgNPs and ZnO-based structures.
(ii) **Preparation of Ag nanoparticles by bioreduction of Ag^+^ in the presence of cloves or mandarin extracts**
  In a volume of cloves extract, an equal volume of 1 mM aqueous solution of AgNO_3_ was added under continuous stirring. Over time, the color of the extract changed from pink to reddish brown, indicating the formation of AgNPs.
  In an adequate volume of mandarin extract, AgNO_3_ crystals were added up to the final concentration of 4 mM under continuous stirring. The change of color from yellow–orange to green–gray was observed, indicating the formation of AgNPs.
  The obtaining of AgNPs was monitored by recording the |Ultraviolet-Visible (UV-Vis) absorption spectra, which shows the appearance of an absorption band in the wavelength region of 400–450 nm, characteristic for the SPR (surface plasmon resonance) band of the AgNPs with the quasi-spherical shape [28].
(iii) **Preparation of ZnO particles by the chemical reaction between Zn (NO_3_)_2_ and NaOH, in the presence of Ag nanoparticles in cloves, respectively mandarins extract**
  The suspension of AgNPs in the cloves or mandarins extract, represents solution A. The precursors: Zn (NO_3_)_2_·6H_2_O and NaOH were dissolved in distilled water (under continuous stirring), obtaining solutions B and, respectively, C. Subsequently solutions B and C were added dropwise into solution A (stirring continued for 30 min). The precipitate was centrifuged and washed several times until a neutral pH was obtained and finally dried in vacuum at 100 °C for 2 h. Additionally, maintaining the same reaction conditions, the ZnO nanoparticles were also obtained in the absence of Ag nanoparticles, in the two extracts: cloves and mandarins. The abbreviations: CUI and MAND correspond to cloves and mandarin derived samples, respectively. The investigated samples are noted as follows: AgZnO-CUI; AgZnO-MAND, ZnO-CUI, ZnO-MAND, Ag-CUI and Ag-MAND.

Synthesis of AgZnO composites involves the following stages:

(i) AgNO_3_ + phyto-molecules (proteins, sugars and phenolic compounds)→Ag^0^
(ii) Ag^0^→AgNPs through the process of capturing Ag^0^ by the extract biomolecules
(iii) Zn(NO_3_)_2_ + 2NaOH→Zn(OH)_2_↓ + 2NaNO_3_
(iv) phyto-AgNPs + ${\mathrm{Zn}(\mathrm{OH})}_{2}\overset{100^{{}^{\circ}}C}{\to }\;\mathrm{phyto-AgZnONPs}+H_{2}O$

The “green” synthesis of ZnO-CUI and ZnO-MAND involves the following steps:

(i) Zn(NO_3_)_2_ + 2NaOH + plant extract →Zn(OH)_2_↓ + 2NaNO_3_ + plant extract
(ii) plant extract + ${\mathrm{Zn}(\mathrm{OH})}_{2}\overset{100^{{}^{\circ}}C}{\to }\mathrm{ZnONPs}\;(\mathrm{capped}\;\mathrm{with}\;\mathrm{phyto-molecules})+H_{2}O$

### 2.2. Physico-Chemical Characterization of Phyto-Developed Materials

FT-IR spectra of the samples were recorded using a Tensor 27 spectrometer Bruker, (Bruker, Billerica, MA, USA), equipped with an attenuated total reflection (ATR) module. Briefly, a volume of 5–10 μL of each sample was placed on the ATR crystal and left to dry before performing the recording. FTIR investigations were performed at room temperature, over the spectral range 400–4000 cm^−1^, with a 4 cm^−1^ spectral resolution and 64 scans per sample. Spectra were corrected for the baseline using the OPUS software and further processing was performed using OriginPro (Microcal Inc., Gyor, Hungary).

UV-Vis spectroscopy was performed using a Varian Cary-100 spectrophotometer (Santa Clara, CA, USA) using 1 cm length quartz cuvette. Absorption spectra were recorded within the 290–800 nm range.

Structural characterization was performed by X-ray diffraction (XRD) using a Bruker D8 Advance diffractometer (Bruker AXS, Karlsruhe, Germany) with CuKα radiation (λ = 0.154 nm). The source was operated at 40 kV and 40 mA, and by using a nickel filter, the Kβ radiation was eliminated. Diffraction patterns were acquired at room temperature in Bragg-Brentano geometry in the range of 2θ from 20° to 80° at a speed of 0.6 °/min (2 θ/min). The XRD data were processed using “Bruker Diffrac plus Basic Package Evaluation v.12”.

The morphology and composition of the samples were investigated using a SEM–EVO 50XVP scanning electron microscope (Carl Zeiss AG, Oberkochen, Germany). 

The analyzed samples were prepared for transmission electron microscopy (TEM) analysis by immersing the powders obtained in ethanol, and the suspension obtained was dropped on a microscopy grid initially deposited with a carbon membrane. TEM was performed using a JEM ARM 200F (Akishima, Tokyo, Japan) analytical transmission electron microscope, at the acceleration voltage 200 kV and equipped with an EDS unit, in order to perform TEM imaging, scanning transmission electron microscopy-high angle annular dark field (HAADF-STEM) imaging and energy-dispersive x-ray (EDX) line analysis spectroscopy. 

### 2.3. Biological Investigation Methods

i. Cell Viability

NPs biocompatibility was evaluated on Human fibroblast BJ cells (ATCC CRL-2522, Manassas, VA, USA) by using MTT (3-(4,5-dimethylthiazol-2-yl)-2,5-diphenyltetrazolium bromide) tetrazolium reduction assay as follows. Cells were seeded in 96-well plates (25,000 cells/well) and cultured for 24 h in MEM (mimimal essential medium) supplemented with 2 mM L-Glutamine, 10% fetal calf serum (FCS) and 100 units/mL of penicillin and 100 µg/mL of streptomycin at 37 °C in a humidified incubator under an atmosphere containing 5% CO_2_. After overnight incubation, the medium was changed and nanoparticles in various concentrations were added for 24 h. As a negative control, cells without any addition were used. Following incubation, the medium was changed and MTT solution was added to each well to a final concentration of 1 mg/mL and incubated for an additional 4 h at 37 °C. Finally, the medium was collected and DMSO was used to dissolve the insoluble formazan product. The absorbance of the samples was recorded at 570 nm using a plate reader Mithras 940 (Berthold, Germany). The data were corrected for the background and the percentage of viable cells was obtained using the following equation: [(A_570_ of treated cells)/(A_570_ untreated cells)] × 100%. The NP concentration that reduced the viability of the cells by half (IC50) was obtained by fitting the data with a logistical sigmoidal equation using the software Origin 8.1 (Microcal Inc., Gyor, Hungary). 

All cell cultivation media and reagents were purchased from Biochrom AG (Berlin, Germany).

ii. Antibacterial Assay

NPs antimicrobial activity was determined against *Staphylococcus aureus* ATCC 6538. Minimum inhibitory concentration (MIC) of the NPs tested was determined by broth microdilution as described previously [29,30]. Bacterial cells were collected in logarithmic phase, washed and suspended at 5 × 10^5^ – 1 × 10^6^ colony-forming units (CFU)/mL in Mueller–Hinton broth (MHB). The NP concentrations were in the range of 0.11–4 mg/mL and in each well it was added 10 µL of bacteria suspension and the plates were incubated under agitation at 35–37 °C for 18–20 h. MICs were determined as the lowest concentration that completely inhibited bacterial growth when examined by eye [29]. All experiments were conducted in duplicate. The minimum bactericidal concentration (MBC) was determined from the wells showing no bacterial growth and incubated further more at 35–37 °C for 18–20 h. The lowest concentration at which no bacterial growth was observed represents the MBC.

iii. Hemolytic Assay

The hemolytic activity of the NPs on human red blood cells (hRBCs) was determined using a protocol adapted from ASTM F 756–00 [31]. Briefly, the blood was collected from volunteers and diluted with phosphate saline buffer (PBS, pH 7.4) to a concentration of hemoglobin of ~10 mg/mL. hRBCs were incubated with various concentrations of NPs for 4 h at 37 °C. Following, the cells were centrifuged, the supernatant collected, transferred into 96-well tissue culture plates, mixed with an equal amount of Drabkin reagent and let to react for 15 min. The samples absorbance was recorded at 570 nm using a plate reader. For negative and positive controls were used human red blood cells (hRBCs) in PBS and distilled water, respectively. The data were corrected for the background, dilution factors and used to calculate the % haemolysis (haemolytic index) according to the following equation: (Absorbance of hemoglobin released by the samples)/(Absorbance of total hemoglobin released) × 100%. Drabkin reagent and standard hemoglobin were purchased from Sigma (Darmstadt, Germany). 

## 3. Results and Discussions

### 3.1. Structural, Morphological and Spectral Characterization of “Green” Synthesized Materials

**Figure 1:**
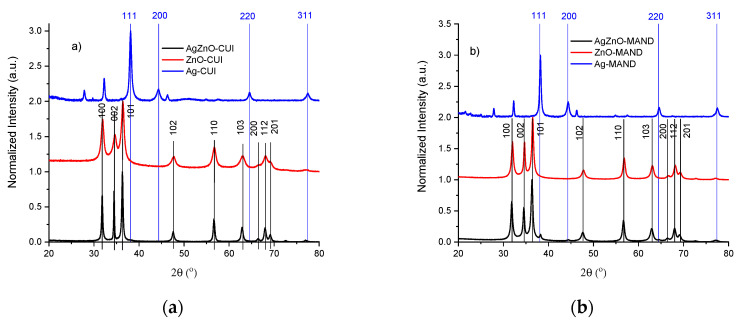
XRD patterns of the samples obtained in (**a**) cloves extract and (**b**) mandarins extract.

The samples were structurally characterized by XRD. The XRD patterns were recorded at room temperature in Bragg-Brentano geometry at an angle of 2θ from 20° to 80°. The XRD patterns obtained on the synthesized samples: AgZnO-CUI, AgZnO-MAND, ZnO-CUI, ZnO-MAND, Ag-CUI and Ag-MAND are shown in Figure 1. The diffractograms of ZnO-CUI and ZnO-MAND show main maxima at 31.8°, 34.7°, 36.3°, 47.6°, 56.6°, 62.9°, 66.4°, 67.9° and 69.1° which correspond to the Miller indices of plans (100), (002), (101), (102), (110), (103), (200), (112) and (201).

The above mentioned diffraction peaks are assigned to ZnO in hexagonal phase of wurtzite (ICDD file no. 01-082-9745) and confirm the obtaining of ZnO particles. The XRD patterns of the AgNPs samples have main maxima at 38.1°, 44.3°, 64.4° and 77.4° which correspond to the Miller indices of the planes (111), (200), (220) and (311) which are attributed to Ag in cubic phase (ICDD file no. 04-001-2617) and confirm the obtaining of AgNPs. In addition to these representative maxima for silver nanocrystals in the XRD patterns related to AgNPs samples, additional peaks were also observed at 27.9°, 32.2°, 46.3°, and 54.8°. These peaks are due to the organic compounds from the extracts and are responsible for reducing the silver ions and stabilizing the resulting nanoparticles [32,33]. The XRD patterns obtained on the composite samples have maxima associated with both Ag and ZnO.

**Figure 2:**
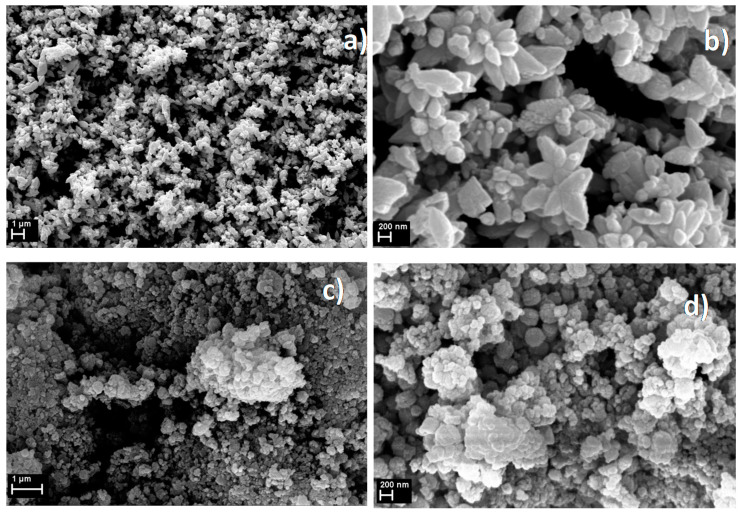

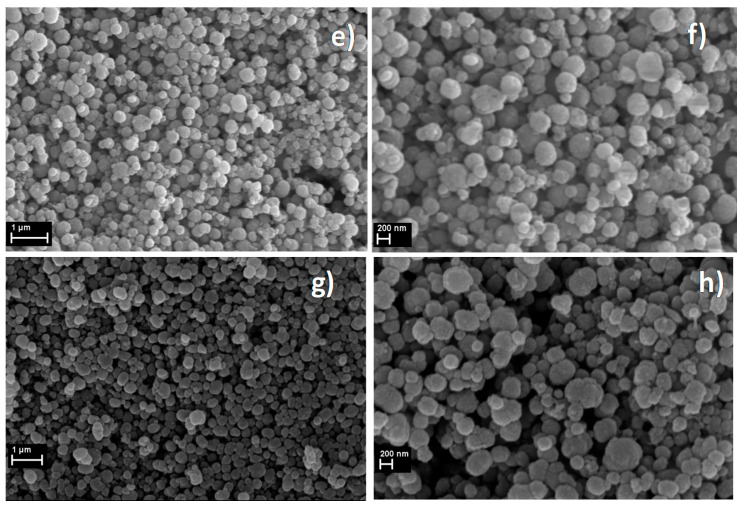
SEM images at two magnifications of samples: (**a**,**b**) AgZnO-CUI; (**c**,**d**) ZnO-CUI; (**e**,**f**) AgZnO-MAND and (**g**,**h**) ZnO-MAND.

The morphology of the phyto-synthesized samples was directly observed by SEM. Figure 2 shows SEM images of the “green” developed structures: AgZnO-CUI, ZnO-CUI, AgZnO-MAND, and ZnO-MAND. The natural extracts play a vital role in the morphological changes of ZnO, as can be seen in Figure 2.

**Figure 3:**
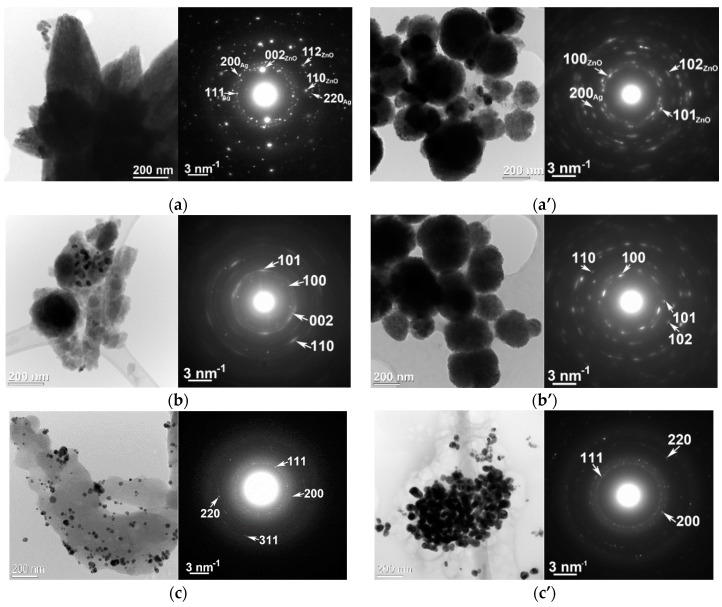
Scanning transmission electron microscopy-high angle annular dark field (HAADF-STEM) images and SAED diagrams for (**a**) AgZnO-CUI, (**b**) ZnO-CUI, (**c**) Ag-CUI, (**a’**) AgZnO-MAND, (**b’**) ZnO-MAND and (**c’**) Ag-MAND.

Additionally the structure and morphology were investigated by TEM measurements (Figure 3), and the results confirm the XRD and SEM data.

The SEM (Figure 2) and TEM (Figure 3) images highlight the morphology of the ZnO particles as self-assembled star-like structures in the case of the AgZnO-CUI sample decorated with AgNPs, while in the case of the AgZnO-MAND sample, spherical particles of ZnO decorated with AgNPs were obtained. For pure ZnO-CUI and ZnO-MAND samples, the SEM and TEM images highlight the ZnO particles in spherical form but also areas without any specific morphology, for the ZnO-CUI sample. The morphology of the structures obtained for the ZnO-MAND sample shows only spherical ZnO particles. The corresponding SAED (Selected Area Electron Diffraction) diagrams (Figure 3) also attest that ZnO has a hexagonal structure with the P63mc space group (186) in both composite and pure samples, and AgNPs have a cubic structure with the Fm3m space group (225).

The TEM image for AgZnO-CUI and AgZnO-MAND samples (Figure 3a,a’ left) at low magnification evidence the morphology of ZnO particles as self-assembled star-type structures, and some silver nanoparticles can also be observed for the AgZnO-CUI sample. For AgZnO-MAND, the TEM image reveals ZnO spherical particles with an average size ranging from 50 to 400 nm, and among them some AgNPs. The SAED diagrams corresponding to the TEM images (Figure 3a,a’ right), including AgNPs, show the two crystalline structures, hexagonal ZnO with the space group P63mc (186) and cubic Ag with the space group Fm-3m (225). The low magnification TEM image (Figure 3c left), corresponding to sample Ag-CUI, shows a more homogeneous distribution of Ag particles as opposed to sample Ag-MAND (Figure 3c’ right), which are smaller in size and embedded in an amorphous matrix. Electron diffraction pattern (Figure 3c right) does not differ too much from the case of Ag-MAND, highlighting the cubic structure.

For sample ZnO-CUI, the low magnification TEM image (Figure 3b left) shows the morphology of the ZnO particles as a mixture of spherical particles and areas without any particular morphology. In the case of the ZnO-MAND sample, unlike the ZnO-CUI sample, the TEM image at low magnification (Figure 3b’ left) shows only spherical ZnO particles embedded in an amorphous matrix. The electron diffraction diagram (Figure 3b right) corresponding to the TEM image, highlights the hexagonal structure of ZnO with the space group P63mc (186) without any other secondary phase. The electron diffraction diagram (Figure 3b’ right) corresponding to the TEM image, highlights the hexagonal structure of ZnO, without any other secondary phase. Unlike the previous case (ZnO-CUI), a high intensity of the diffraction rings can be observed due to a higher crystallinity of the ZnO particles. The low magnification TEM image (Figure 3c left), corresponding to sample Ag-CUI, shows a more homogeneous distribution of Ag particles as opposed to sample Ag-MAND (Figure 3c’ right), which is smaller in size and embedded in an amorphous matrix. Electron diffraction pattern (Figure 3c,c’ right) highlights the cubic structure of AgNPs.

**Figure 4:**
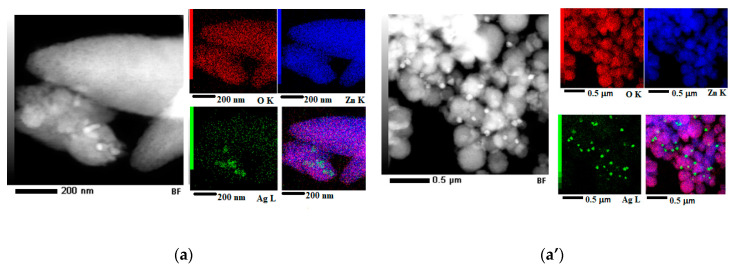

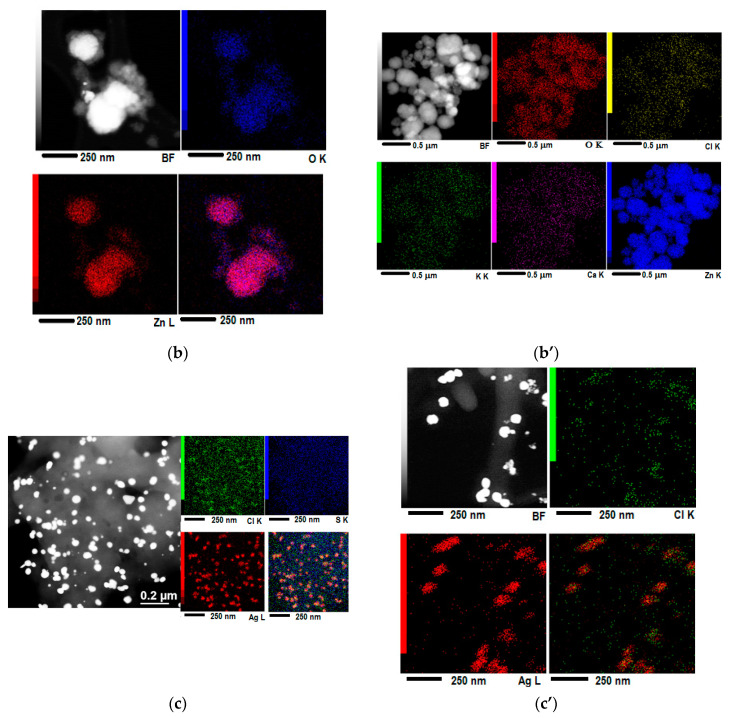
HAADF-STEM image at low magnification and elementary maps obtained by EDS mapping corresponding to the HAADF-STEM image, for (**a**) AgZnO-CUI (Ag L—green, Zn K—blue and O K—red), (**b**) ZnO-CUI (Zn K—red and O K—blue), (**c**) Ag-CUI (Ag L—red, Cl K—green, S K—blue), (**a’**) AgZnO-MAND (Ag L—green, Zn K—blue and O K—red), (**b’**) ZnO-MAND (O K—red, Cl K—yellow, K K—green, Ca K—purple and Zn K—blue) and (**c’**) Ag-MAND (Ag L—red and Cl K—green). For each sample, the last image was obtained by overlapping the individual elemental maps.

EDS mapping (Figure 4a right), performed on an area from the HAADF-STEM image (Figure 4a left), highlights the distribution of O, Zn and Ag elements. Elemental maps confirm the presence of AgNPs (see Ag L-green map) as agglomerations of nanoparticles on the surface of ZnO structures (see Zn K-blue and O K-red map) for the AgZnO-CUI sample. For ZnO-CUI, the EDS mapping (Figure 4b right) shows the homogeneous distribution of Zn and O elements in the HAADF-STEM image (Figure 4b left). Additionally, the EDS mapping for the Ag-CUI sample (Figure 4c right) shows the Ag distribution on the analyzed area (see HAADF-STEM image Figure 4c left), as well as the appearance of Cl and S elements (see Cl K-green map, S K-blue) belonging to the amorphous area which incorporates AgNPs. 

Using EDS mapping, the distribution of the elements from the analyzed area corresponding to the STEM image was obtained for AgZnO-MAND (Figure 4a’). Unlike the first sample, AgZnO-CUI, in the HAADF-STEM image (Figure 4a’ left) a more homogeneous dispersion of AgNPs is observed on the surface of ZnO spheres, this being confirmed by the EDS RGB map (Figure 4a’ lower right) obtained by overlapping the maps Ag L—green, Zn K—blue and O K—red. The EDS mapping (Figure 4b’ right) made on the HAADF-STEM image of ZnO-MAND sample (Figure 4b’ left) shows the distribution of the Zn and O elements as well as the presence of some additional elements such as Cl, K and Ca. The corresponding EDS maps (see Figure 4b’ Cl K—yellow, K K—green, Ca K—purple) indicate the origin of these elements as being from the amorphous matrix that encloses the ZnO particles. For the Ag-MAND sample, the EDS mapping (Figure 4c’) shows the Ag distribution on the analyzed area (see HAADF-STEM image Figure 4c’ left), as well as the appearance of Cl element (see Cl K—green map) in the areas corresponding to the porous structures.

**Figure 5:**
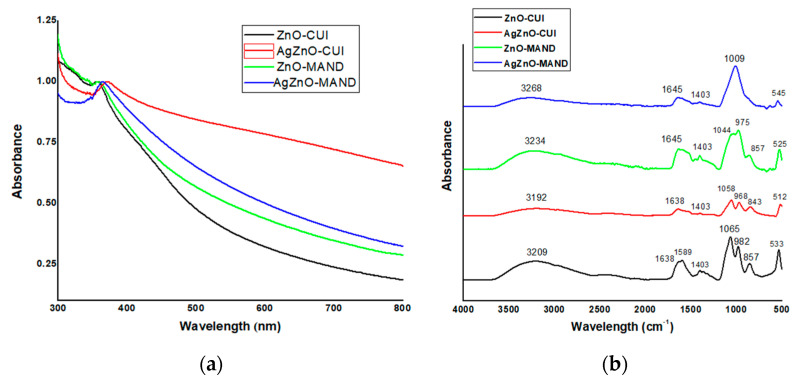
Optical characterization of “green” developed materials: (**a**) UV-Vis absorption spectra (normalized at the ZnO corresponding UV absorption peak) and (**b**) FTIR spectra of the phytosynthesized materials.

UV-Vis absorption spectroscopy (Figure 5a) was also performed to confirm the formation of ZnO-based composites. The spectral fingerprint of ZnO nanoparticles [34] was observed at 357 nm, 371 nm, 358 nm and 364 nm for ZnO-CUI, AgZnO-CUI, ZnO-MAND and AgZnO-MAND, respectively. UV-Vis spectra indicated that the presence of AgNPs into ZnO-based materials resulted in a “red” shift in the absorption peak characteristic for ZnO nanoparticles, due to surface modification of ZnO particles, confirming the development of AgZnO composites. 

Figure 5b displays the comparative presentation of FT-IR spectra for phyto–derived materials designed in this study, and in Table S1 (Supplementary Material) are shown the assignments of the main FT-IR bands.

The broad band centered in the range 3268–3192 cm^−1^ confirmed the presence on the composites’ surface of hydroxyl groups belonging to polyphenols and polysaccharides originating from vegetal extracts. Other main FTIR bands were observed at 1638–1589 cm^−1^, 1403 cm^−1^, 1065–968 cm^−1^, 857–843 cm^−1^ assigned to amide I (carbonyl (–C=O) stretch in proteins) and carboxylate (–COO–) groups, phenol O–H bend, ether (–C–O–C–) groups of polysaccharides, hydrogen-bonded O–H out-of-plane bending, respectively; these functional groups belong to biomolecules (proteins, carboxylic acids, polysaccharides, polyphenols) arising from plant extracts. The presence of FT-IR bands in the range 545–512 cm^−1^ confirmed the development of ZnO-based nanostructures. 

### 3.2. Biological Characterization of Phytosynthesized Composites

Finally, the biological properties such as, antibacterial activity, cytotoxicity/biocompatibility, hemolytic activity and therapeutic index of phytosynthesized samples were analyzed.

The antibacterial effects of the samples were tested against the most common nosocomial pathogen *Staphylococcus aureus*, and the values obtained for MIC and MBC are presented in Table 1. 

The antibacterial behavior of the obtained composites is explained by their composition based on organic (rich in polypenolic compounds arising from vegetal extracts) and inorganic (Ag, ZnO) components possessing antibacterial activity, giving a synergic action against tested pathogen. Moreover, research studies have demonstrated that both AgNPs and ZnONPs destroy the microbes by a mechanism that involves the main following steps: (1) cell wall damaging of the pathogen and penetration inside the microorganism; (2) cell membrane disrupting; (3) nanoparticle (NPs) internalization and interference with metabolic functions of microbes causing their death [2]. The most effective sample proved to be AgZnO-CUI with an MIC value of 0.11 mg/mL and an MBC value of 2.68 mg/mL. All the other three composites were inhibiting bacterial growth at a concentration between 0.25 mg/mL and 0.37 mg/mL with a bactericidal concentration between 3 mg/mL and 4 mg/mL. 

Our composites presented better antibacterial action than the Ag–ZnO nanocomposites reported by previous studies. Nguyen et al. reported an inhibitory action against *S. aureus* at 8 mg/mL [35], while Lu et al. [36] reported MIC values for Ag–ZnO nanocomposites at 0.4 mg/mL against *S. aureus*. 

The strong antibacterial behavior of AgZnO-CUI is due to its morphology with randomly oriented spatial star-like structures containing edges and corners that cause lethal bacterial cell injury, more than regularly arranged structures [37]. Thus, the cell wall and cell membrane are damaged and AgZnO-CUI particles could be then easily internalized in the pathogen cell. The internalization mechanism depends on particle morphology. Talebian et al. showed that flower-shaped ZnO-NPs exhibited higher biocidal activity against *S. aureus*, and *E. coli* than the spherical-shaped ZnO-NPs [38]. In addition, AgZnO-CUI composition includes the AgNPs generated using cloves’ extract which possess strong antibacterial properties [14]. 

**Figure 6:**
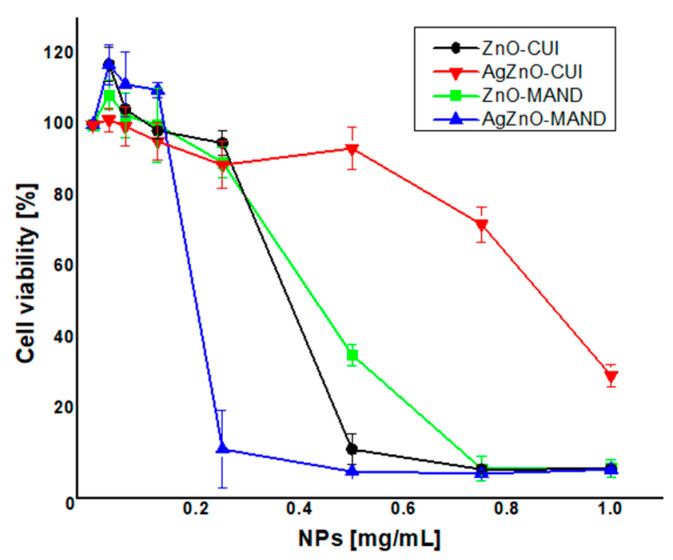
Cytotoxic effect of the samples on viability of BJ cells evaluated at 24 h using MTT assay.

The biocompatibility of the samples was tested against BJ cells for concentrations between 0.012 and 1 mg/mL, which are representative for the concentrations where MIC values were detected. The results are presented in Figure 6.

ZnO-MAND and ZnO-CUI samples showed a similar behavior against BJ cells, decreasing cells’ viability in a dose dependent manner with increasing concentrations of the NPs. Their IC_50_ values are 0.43 mg/mL for ZnO-MAND and 0.35 mg/mL for ZnO-CUI. AgZnO-MAND proved to be more toxic than ZnO-MAND, reducing the cell viability at ~10% for concentrations higher than 0.2 mg/mL. The IC_50_ value of AgZnO-MAND is 0.22 mg/mL, two times smaller compared to IC_50_ of ZnO-MAND. The most efficient NPs were AgZnO-CUI. For them, cell viability becomes affected at concentrations higher than 0.6 mg/mL and it decreases at ~30% for the highest concentration tested. When calculated, IC_50_ was 0.9 mg/mL, almost three times higher than the one of ZnO-CUI. Based on these results we can say that from the four obtained NPs, AgZnO-CUI is less toxic for eukaryotic cells.

Table 1 demonstrates the minimal hemolytic concentration (MHC) for samples tested. All the samples tested did not induce any hemolysis for concentrations between 0–1 mg/mL. 

The therapeutic index (TI) is used to report cell specificity of different pharmacological agents [39,40]. TI was calculated as the ratio of the values that produce a toxic effect (min (MHC, IC_50_)) over MIC (Table 1). For the highest concentration tested that did not induce any cytotoxic/hemolytic activity, a value two times higher was used to calculate the therapeutic index [41]. A TI higher than one is indicative of a higher specificity of the NPs for the bacterial cells [39]. The highest TI was obtained for AgZnO-CUI, with a value of 8.18, followed by ZnO-MAND with a TI of 1.72. The least effective was ZnO-CUI with a TI of 0.95, followed by AgZnO-MAND, with a TI value of 0.65. AgZnO-CUI proved to be the most efficient antibacterial agent against *S. aureus* bacteria with reduced toxicity against eukaryotic cells at concentrations smaller than 0.36 mg/mL. 

## 4. Conclusions

This study described “green” routes to build up novel phyto-based composites with great significance for biomedical fields. Two types of vegetal extracts (from cloves and mandarin peels) were used as ecological bioreducing agents to achieve four kinds of metallic composites. 

The characteristics of the “green” synthesized composites were evaluated by microscopy (SEM and TEM) images, and spectral investigations (EDS, XRD, UV-Vis absorption and FT-IR spectroscopy). EDS maps showed the presence of phytogenic silver nanoparticles on surface of ZnO nanostructures. 

From all the obtained materials, AgZnO-CUI showed a good biocompatibility (on human fibroblast BJ cells), no damage the human red blood cells and improved bactericidal action against the most common nosocomial pathogen, *Staphylococcus aureus*. The improved efficacy is due to presence of silver in Ag-loaded ZnO materials. AgZnO-CUI composite can become a good candidate for biomedical studies. 

Therefore, the “green” synthesis of AgZnONPs structures is an efficient way to build composites with improved antibacterial properties. 

## Figures and Tables

**Table 1:** Bacteriostatic (MIC) and bactericidal (MBC) action against *S. aureus*; the half viability concentration (IC_50_) on BJ cells, minimal hemolytic concentration (MHC) and calculated TI for tested NPs.

Sample	MIC (mg/mL)	MBC (mg/mL)	MHC (mg/mL)	IC_50_ (mg/mL)	TI
ZnO-CUI	0.37	3.4	>1	0.35	0.95
AgZnO-CUI	0.11	2.68	>1	0.90	8.18
ZnO-MAND	0.25	4	>1	0.43	1.72
AgZnO-MAND	0.34	2.93	>1	0.22	0.65

MIC—minimal inhibitory concentration; MBC—minimal bactericidal concentration; MHC—minimal hemolytic concentration is considered according to the ASTM F 756-00 as the concentration at which the samples induce a 5% hemolysis; TI (Therapeutic index) = min (MHC, IC_50_, LDH50)/MIC. When no toxic activity was detected at the highest concentration tested, for TI calculation was used a value two times higher. Larger values indicate greater cell selectivity.

## Supplementary Materials

The following are available online at https://www.mdpi.com/2079-4991/10/11/2146/s1, Table S1. FT-IR bands’assignment for phyto-derived materials synthesized in this study.
