# Supplementary material for: Performant Composite Materials Based on Oxide Semiconductors and Metallic Nanoparticles Generated from Cloves and Mandarin Peel Extracts
Source: Nanomaterials (Basel). 2020 Oct 28;10(11):2146. doi: 10.3390/nano10112146 (PMC7693827; doi:10.3390/nano10112146)
Supplement: Supplementary file 1 [file nanomaterials-10-02146-s001.pdf]

nanomaterials

Supplementary Materials

Performant Composite Materials Based On Oxide Semiconductors and Metallic Nanoparticles Generated from Cloves and Mandarin Peel Extracts

Irina Zgura [1,*], Monica Enculescu [1], Cosmin Istrate [1], Raluca Negrea [1], Mihaela Bacalum [2], Liviu Nedelcu [1] and Marcela Elisabeta Barbinta-Patrascu [3,*]

[1] National Institute of Materials Physics, Atomistilor 405A, 077125 Magurele, Romania; mdatcu@infim.ro (M.E.); cosmin.istrate@infim.ro (C.I.); raluca.damian@infim.ro (R.N.), nedelcu@infim.ro (L.N.)
[2] Horia Hulubei National Institute for Physics and Nuclear Engineering (IFIN-HH), Department of Life and Environmental Physics, 077125 Bucharest-Magurele, Romania; bmihaela@nipne.ro
[3] Faculty of Physics, University of Bucharest, 405 Atomistilor Street, P.O. Box MG-11, 077125 Bucharest-Magurele, Romania
* Correspondence: irina.zgura@infim.ro (I.Z.); elipatras@gmail.com (M.E.B.-P.)

Table S1. FT-IR bands' assignment for phyto-derived materials synthesized in this study.

Sample	FT-IR Bands (cm^{-1})	Attribution	Ref.
ZnO-CUI / AgZnO-CUI	3209 (intense, broad)/3192 (broad)	O–H bending and stretching vibrations in phenolic compounds, alcohols, and polysaccharides<br>N–H stretching vibrations (peptides and proteins)	[1, 2]
	1638; 1596/ 1638; 1525	Amide I, due to –C=O stretch in proteins<br>Carboxylate (–COO–) groups	[3, 4]
	1403	O–H bend (phenol or tertiary alcohol)	[4]
	1065; 982/ 1058; 968	Antisymmetric stretching of C–O group of polysaccharides, alcohols	[4, 5]
	857/ 843	Hydrogen-bonded O–H out-of-plane bending	[4]
	533/512	Zn-O stretching vibration	[6]
ZnO-MAND/ AgZnO-MAND	3234/3268 (very broad band)	The hydrogen-bonded O–H (bending and stretching vibrations) groups in phenolic compounds and in polysaccharides like pectin<br>N–H stretching vibrations (peptides and proteins)	[2, 4, 7, 8]
	1645, 1528	Carboxylate groups (–COO–) present in aminoacids or in pectin derived from mandarin peels<br>Amide I, arising from –C=O stretch in proteins	[4]
	1403 (weak)	Phenol or tertiary alcohol, O–H bend	[4]
	1044, 975/ 1009 (sharp band)	Stretching vibration of –C–O–C– ether linkage groups of polysaccharides (pectin<br>ν (CO), ν (CC) ring of polysaccharides (like pectin)	[4, 9]
	857	Hydrogen-bonded O–H out-of-plane bending	[4]
	525/545 (sharp)	Zn-O stretching vibration	[6]

© 2020 by the authors. Submitted for possible open access publication under the terms and conditions of the Creative Commons Attribution (CC BY) license (http://creativecommons.org/licenses/by/4.0/).

---

1. Barbinta-Patrascu, M.E.; Badea, N.; Bacalum, M.; Ungureanu, C.; Suica-Bunghez, I.R.; Iordache, S.M.; Pirvu, C.; Zgura, I.; Maraloiu, V.A. 3D hybrid structures based on biomimetic membranes and Caryophyllus aromaticus-"Green" synthesized nano-silver with improved bioperformances. *Mater. Sci. Eng. C* **2019**, *101*, 120–137.
2. Estrada-Urbina, J.; Cruz-Alonso, A.; Santander-González, M.; Méndez-Albores, A.; Vázquez-Durán, A. Nanoscale Zinc Oxide Particles for Improving the Physiological and Sanitary Quality of a Mexican Landrace of Red Maize, *Nanomat*. **2018**, *8*, 247.
3. Barbinta-Patrascu, M.E.; Ungureanu, C.; Iordache, S.M.; Iordache, A.M.; Bunghez, I.R.; Ghiurea, M.; Badea, N.; Fierascu, R.C.; Stamatin, I. Eco-designed biohybrids based on liposomes, mint—nanosilver and carbon nanotubes for antioxidant and antimicrobial coating. *Mat. Sci. Eng. C* **2014**, *39*, 177–185.
4. Coates, J. *Interpretation of Infrared Spectra, a Practical Approach. In Encyclopedia of Analytical Chemistry*; Meyers, R.A., Eds.; John Wiley & Sons Ltd: Chichester, UK, 2000.
5. Balachandran, Y.L.; Girija, S.; Selvakumar, R.; Tongpim, S.; Gutleb, A.C.; Suriyanarayanan, S. Differently Environment Stable Bio-Silver Nanoparticles: Study on Their Optical Enhancing and Antibacterial Properties. *PLoS ONE* **2013**, *8*, e77043.
6. Jankovic, S.; Milisavic, D.; Okolic, T.; Jelic, D. Synthesis of ZnO-Ag nanoparticles by sol-gel method, *Contemporary Materials*, **2019**, *X*, 22-27.
7. Kanmani, P.; Dhivya, E.; Aravind, J.; Kumaresan, K. Extraction and Analysis of Pectin from Citrus Peels: Augmenting the Yield from Citrus limon Using Statistical Experimental Design. *Iran. J. Energy Environ*. **2014**, *5*, 303–312.
8. Barbinta-Patrascu, M. E.; Constantin, M.; Badea, N.; Ungureanu, C.; Iordache, S. M.; Purcar, V.; Antohe, S. Tangerine-generated silver-silica bioactive materials *Rom. J. Phys.* **2019**, *64*, 701.
9. Barbinta-Patrascu, M.E.; Badea, N.; Ungureanu, C.; Iordache, S.M.; Constantin, M.; Purcar, V.; Pirvu, C.; Rau, I. Eco-Biophysical Aspects on Nanosilver Bio-Generated from Citrus reticulata Peels, as Potential Bio-Pesticide for Controlling Pathogens and Wetland Plants in Aquatic Media. *J. Nanomater*. **2017**, *2017*, 4214017.
